# Considerations for defining +80 Da mass shifts in mass spectrometry-based proteomics: phosphorylation and beyond

**DOI:** 10.1039/d3cc02909c

**Published:** 2023-09-06

**Authors:** Leonard A. Daly, Christopher J. Clarke, Allen Po, Sally O. Oswald, Claire E. Eyers

**Affiliations:** a Centre for Proteome Research, Department of Biochemistry and Systems Biology, Institute of Systems, Molecular and Integrative Biology, University of Liverpool Liverpool L69 7ZB UK CEyers@liverpool.ac.uk

## Abstract

Post-translational modifications (PTMs) are ubiquitous and key to regulating protein function. Understanding the dynamics of individual PTMs and their biological roles requires robust characterisation. Mass spectrometry (MS) is the method of choice for the identification and quantification of protein modifications. This article focusses on the MS-based analysis of those covalent modifications that induce a mass shift of +80 Da, notably phosphorylation and sulfation, given the challenges associated with their discrimination and pinpointing the sites of modification on a polypeptide chain. Phosphorylation in particular is highly abundant, dynamic and can occur on numerous residues to invoke specific functions, hence robust characterisation is crucial to understanding biological relevance. Showcasing our work in the context of other developments in the field, we highlight approaches for enrichment and site localisation of phosphorylated (canonical and non-canonical) and sulfated peptides, as well as modification analysis in the context of intact proteins (top down proteomics) to explore combinatorial roles. Finally, we discuss the application of native ion-mobility MS to explore the effect of these PTMs on protein structure and ligand binding.

## Abstract 2

This feature article discusses mass spectrometry-based strategies for the characterisation, localisation and differentiation of phosphorylation and sulfation on proteins, and considers future directions in the field.

## Graphical abstract

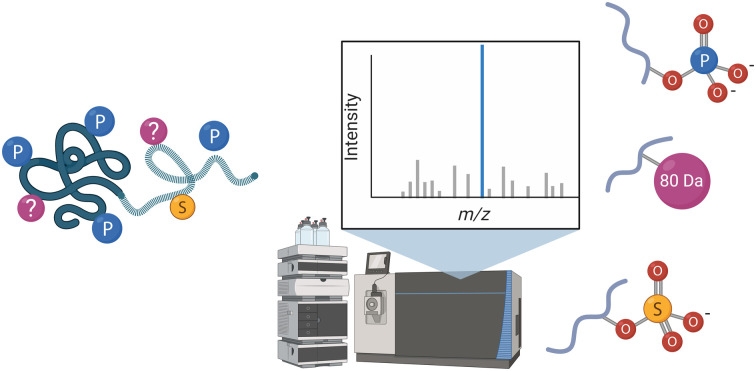

## Introduction

**Fig. 1:**
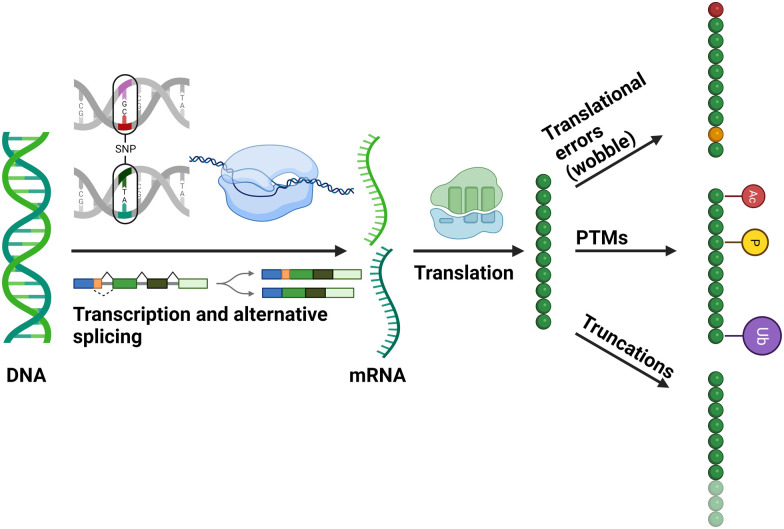
Diagrammatic representation of cellular mechanisms that contribute to the generation of different proteoforms. Figure created with BioRender.com.

Post-translational modification (PTM) is a term used to describe the processing of proteins post synthesis, either through cleavage, covalent attachment of a functional group, or modification of an amino acid side chain. Over 400 biological PTMs have so far been described,^1^ with our knowledge of the diversity of protein modifications continuing to expand. PTMs serve to regulate protein function in a dynamic and often reversible manner and are typically enzyme-driven processes. Covalent PTMs, which will be the focus of this article, are highly diverse, ranging from appendment of relatively small chemical moieties (such as phosphate) to the attachment of complex, whole (poly)protein chains such as ubiquitin. Alone, or in combination, PTMs are essential for the regulation of protein function across all organisms, allowing a rapid physiological response to a change in environment, and negating the need for energetically demanding and comparatively slow *de novo* synthesis. PTMs also diversify the available functional proteome, generating millions of (potentially functionally different) protein species from a defined number of genes. While the human genome only encodes for ∼21 000 genes, it has been estimated that in excess of 6 000 000 proteoforms (expressed gene products with different combinations of single nucleotide polymorphisms and/or PTMs) can be generated (Fig. 1).^2–4^

The effect of a PTM is dependent on the type and site of the modification on a given protein, with wide-ranging functional consequences, such as sub-cellular shuttling, altering a protein's ability to bind to co-factors, other proteins or nucleic acids, regulation of enzyme activity and altered stability.^5–7^ Errors, or mis-regulation in protein PTMs consequently changes the ability of a system to adapt to its environment and can result in disease.^8–11^ Thus, defining the ‘what’ and ‘where’ of PTMs on a protein is essential to understanding the ‘why’, and the ‘what went wrong’.

Mass spectrometry (MS) has become the ‘gold standard’ for PTM analysis, capable of both targeted analysis of proteins (and PTMs) of interest, and large scale, high throughput (HTP) PTM studies and the investigation of signalling networks. As well as being faster and less biased than more traditional biochemical approaches (*e.g.* antibodies or radioactive labelling), MS is able to define the type of PTM, pinpoint its location (something that typically requires validated site-specific antibodies or Edman degradation of a radiolabelled product) and benefits from being (semi)quantitative, thus allowing PTM dynamics in a changing environment to be evaluated. MS has thus revolutionised the interrogation of the proteome and PTM-mediated signalling cascades.

For the last 15 years, our group has developed MS-based analytical strategies for the identification and quantification of PTMs with a view to improving our understanding of signalling biology. In this feature article, we discuss some of our recent developments placing them in the broader context of the field, focussing in particular on those PTMs that induce a +80 Da mass shift – phosphorylation and sulfation. We also explore avenues for potential future investigation to improve our understanding of these covalent modifications.

## From single protein analysis to high-throughput characterisation

One of the most common PTMs is protein phosphorylation, namely the reversible and covalent addition of a phosphate group (HPO_3_^−^) to the side chain of amino acids, predominantly (albeit not exclusively) the alcohol-containing side chain of Ser, Thr and Tyr residues.^12–18^ Certainly, the vast majority of studies investigating the role of phosphorylation to date have focused on these three residues and have been key to advancing our understanding of the critical roles that this PTM plays in mammalian cell signalling (and their dysregulation in disease). There are a number of comprehensive reviews covering this topic^19,20^ and will not be the primary focus of this article.

Our contributions to understanding the biological roles of specific protein phosphorylation (and other PTMs) events involve: characterising and exploring the effects of novel sites on numerous protein kinases, including the cell cycle-regulated enzymes Aurora A and PLK4,^21–26^ quantifying dynamic site-specific phosphorylation with demonstrable effects on catalytic activity. We have also explored the roles of specific phosphosites on differential dimerization and DNA binding ability of transcription factors such as the NF-κB subunit RelA and hypoxia-inducible factor (HIF).^27–29^ Of note, in-depth characterisation of proteins (by our group and others) inevitably reveals that a protein is typically decorated by multiple sites and types of PTM, often with numerous co-occurring sites of phosphorylation. Current estimates suggest that a third of human proteins are phosphorylated at any one time, and that ∼90% of all proteins are capable of being phosphorylated at some point in their life-cycle. This is supported by the fact that 17.8k of the ∼20k proteins in the human UniProt (reviewed) database are defined as being phosphorylated in PhosphoSite Plus (PSP).^13,30–32^ Consequently, there has been a move away from focused investigations of specific proteins of interest to large-scale HTP phosphorylation site mapping to interrogate signalling networks.

The rise of HTP phosphopeptide characterisation studies (phosphoproteomics) over the last 20 years means that databases such as PSP and Peptide Atlas (PA) are awash with ‘identified’ sites. However, variance in the confidence with which these phosphorylation sites have been identified means that many of these sites are unlikely to be real (either due to mislocalisation or poor spectral matching); while PSP currently lists over 130k unique phosphosites, PA details 164k sites, of which only 60% are shared between both databases.^32^ Considering multiple observations in HTP studies, as well as orthogonal features such as evolutionary conservation, contextual information and functional enrichment analysis, we conservatively estimate that only ∼82k of these are likely to be real,^32^ hence databases of phosphorylation sites (and other PTMs) should be used with a degree of caution.

There is no doubt that we still have a long way to go to fully define the human phosphoproteome, let alone the functional significance or regulation of specific sites. Moreover, taking a wider perspective, deciphering phosphorylation sites in other species remains a substantial challenge. Beyond incomplete proteome databases, signalling in plants, fungi and prokaryotes is extensively regulated through phosphorylation of Asp and His residues as part of the two-component systems.^33^ Numerous other amino acids can also be covalently modified by phosphate,^16–18,34^ significantly expanding the search space complexity, and thus the rate of mis-localisation.

## The need for phosphopeptide enrichment

Protein phosphorylation, like other types of PTM, is seldom stoichiometric. Consequently, any analytical strategy looking to characterise PTMs must consider their comparatively low abundance compared with non-modified peptides generated from complex biological samples. Thus, methods have been developed to enrich a specific type of PTM, by invoking PTM-specific biochemical properties, prior to HTP MS analysis. In the case of phosphopeptides, the low p*K*_a_ value of phosphate (primary p*K*_a_ of phosphate monoesters: ∼2) *versus* acidic aspartic acid and glutamic acid side chains (p*K*_a_: ∼3.5 and ∼4.2 respectively)^35^ is often used to facilitate the efficient and (relatively) specific enrichment of phosphopeptides by manipulating pH.

There are two major phosphopeptide enrichment strategies currently in play: Metal Oxide Affinity Chromatography (MOAC) and Immobilised Metal ion Affinity Chromatography (IMAC), which have similar, but fundamentally different, approaches for phosphate capture.^36^ MOAC, most commonly titanium dioxide, relies on the specific spatial properties of phosphate moieties and efficient bidentate hydrogen bonding to immobilised titania for enrichment.^37–40^ IMAC, most commonly titanium^4+^ and iron^3+^ (although many other metal ions have been investigated), relies on the much lower p*K*_a_ of phosphate (relative to Asp and Glu) for the specific electrostatic interaction with immobilised metal ions for phosphate capture, with strategies such as methyl esterification or the inclusion of glycolic acid having been used to improve specificity.^21,41–43^ Both phosphorylation enrichment strategies encompass their own select benefits and caveats, however, a detailed discussion is beyond the scope of this review and has been presented elsewhere.^36,44^

## HTP phosphorylation analysis is more than just peptide identification

To explore the (patho)physiological roles of specific phosphosites, confidence in phosphorylation site localisation is essential. The ability to localise phosphorylation sites on peptides during HTP MS/MS has been evaluated using a variety of different fragmentation regimes and software tools. Table 1 provides an overview of the advantages and disadvantages of different fragmentation strategies for the identification of phosphorylated (and sulfated) peptides. A detailed overview of phosphopeptide fragmentation mechanisms is outside the scope of this article, but has been reviewed by Potel *et al.*^45^ The most common peptide ion fragmentation regimes are collision-induced, being either resonant collisional-induced dissociation (performed in ion traps, referred to as resonant CID) and higher energy, non-resonant CID as performed in a quadrupole or octopole-based collision cell (referred to as (beam-type) CID or HCD (in the family of instruments from Thermo Scientific)). However, it is well documented that phosphopeptides subjected to (resonant) CID undergo extensive neutral loss of the phosphate group (*Δ* 80 amu) with/without the additional loss of H_2_O (*Δ* 98 amu), depending on the phosphorylated residue,^12,46–48^ thus hampering the ability to pinpoint the site of phosphorylation on the peptide backbone.^49–55^ Inevitably, site localisation becomes increasingly problematic as the number of potential phosphorylation sites on a given peptide increases. Extensive investigation has revealed that a variety of factors influence the degree of phosphopeptide neutral loss, primarily revolving around the number, and relative position, of basic residues. Specifically, if the charge state of a phosphopeptide ion is greater than the number of basic residues (Arg, Lys, His) resulting in a ‘mobile proton environment’ (MPE), there is less neutral loss.^46,47,53–57^ Contributing to the understanding of the MPE effect on phosphopeptide fragmentation by CID, we demonstrated that promoting a MPE through removal of the C-terminal Arg/Lys residue (which acts as a proton ‘sink’) from tryptic phosphopeptides with carboxypeptidase-B (CBP-B) markedly reduced neutral loss and increased peptide backbone fragmentation, consequently improving both the number and confidence in phosphopeptide identifications.^58^ While this study was interesting from a mechanistic perspective, the subsequent reduction in overall peptide ion charge state (reducing protein coverage due to an increase in singly protonated species) combined with the wider accessibility of different fragmentation regimes now available means that this approach likely has limited utility for HTP phosphoproteomics. However, the use of CBP-B for removal of C-terminal basic residues could be interesting to explore in the context of bigger peptides generated with LysC/ArgC. A larger scale study looking at over 34k publicly available CID-generated MS/MS spectra suggests that for peptide ions with a MPE, neutral loss propensity inversely correlated with phosphate distance (3–8 residues) from the N-terminus, and was reduced for peptides with acetylated N-termini, suggesting direct involvement of the N-terminal amine group in the loss of H_3_PO_4_.^59^ Interestingly, the presence of a proline within the peptide appeared to reduce neutral loss irrespective of its position relative to the site of phosphorylation; this is likely due to the decreased flexibility of Pro-containing peptides and thus their reduced ability to form cyclic structures that facilitate charge solvation/bring the phosphate group into proximity with the mobile proton.^59^

**Table 1:** Advantages and disadvantages of different fragmentation strategies for the mass spectrometric analysis of phosphopeptides

Fragmentation type	Advantages	Disadvantages
Resonant CID	• Fast	• Extensive neutral loss compromises localisation of labile PTMs (phosphorylation, sulfation)
	• No internal product ions – simpler spectra	

Beam-type CID/HCD	• Fast	• Neutral loss can compromise localisation of labile PTMs (phosphorylation, sulfation)
	• Good sequence coverage	

ETD	• Retention of labile PTMs (including phosphate/sulfate)	• Slow
	• Beneficial for peptides containing multiple sites of phosphorylation/sulfation	• Charge state dependency (not useful for peptides of *z* < 3)
		• Charge reduction, as opposed to fragmentation/dissociation decreases sensitivity of product ion generation
		• Supplemental activation required to generate useful product ions (and overcome ETnoD)

EThcD	• ETD-generated product ions improve site localisation	• Slower (than ETD)
	• Overcomes the charge-state dependency of ETD	• Statistical considerations for confident identification/localisation given the generation of multiple product ion types
	• Multiple fragment ion types increases confidence in identification and site localisation	

UVPD	• Potentially tuneable for optimal fragmentation of target peptides (on non-commercial instrumentation)	• Slowest
	• Multiple fragment ion types increases confidence in identification and site localisation	• Extensive neutral loss compromises localisation of labile PTMs (phosphorylation, sulfation)
		• Poor efficiency of fragmentation
		• Statistical considerations for confident identification/localisation given the generation of multiple product ion types
		• Optimal fragmentation of modified peptides at 193 nm which is not available on commercial instrumentation

Electron-mediated fragmentation regimes (electron transfer dissociation – ETD; electron capture dissociation – ECD) have gained popularity in phosphoproteomics pipelines due to their ability to overcome the extensive neutral loss of HPO_3_/H_3_PO_4_ typically observed with collisional dissociation.^60^ These strategies are particularly useful for accurate phosphosite localisation in instances where there are multiple potential sites of modification and as the number of sites of modification on a peptide increases. In contrast to CID/HCD, electron-driven fragmentation is not dependent on the re-distribution of thermal energy, rather spontaneously occurring after electron transfer (being described as non-ergodic), allowing labile covalent PTMs to remain attached to the amino acid side chain. However, a major deficit of such fragmentation approaches is that ions of charge state ≤2+ (the predominant species for tryptic phosphopeptides) is poor due to non-dissociative charge reduction which compromises the ability to generate fragment ions, a prerequisite for peptide sequence determination and PTM localisation. Dual fragmentation approaches that apply supplemental collisional dissociation of ETD products (EThcD/ETciD) largely overcome the charge state-dependent fragmentation deficiency of ETD, producing rich tandem mass spectra which contain a mixture of product ion types (b/y & c/z).^61,62^ These approaches have been highly useful for characterising labile PTMs, with multiple benchmarking studies (including from our group) having evaluated the utility of EThcD as the optimal method for correct phosphorylation site localisation.^49,63,64^ Our analyses revealed that the benefits of EThcD for phosphopeptide identification and low probability of site mis-assignment is dependent on the resolution at which MS2 data are acquired, as well as the algorithm used for data interrogation.^49^ Importantly, while we observed a notable increase in the proportion of correctly localised phosphorylation sites for a synthetic phosphopeptide library in low resolution (ion trap; IT) experiments (using an Orbitrap Fusion instrument; 92% for EThcD *versus* 83% for HCD), there was a marked reduction in the number of peptide spectral matches (PSMs) (654 *versus* 1497 respectively) under the same conditions, revealing a ‘double-edged sword’ regarding the utility of EThcD for HTP phosphoproteomics. This reduction in PSMs (and consequently peptide identification rate) is a known downside to ET(hc/ci)D arising from the increased duty cycle of this sequential fragmentation regime. In contrast, there was minimal benefit in phosphosite localisation confidence rates between HCD and EThcD for this phosphopeptide library at high resolution (94% *versus* 96% respectively), although the reduction in PSMs remained substantial (889 *versus* 417 respectively). We originally hypothesised that the relatively limited utility of EThcD observed in this benchmarking study may in part be due to the composition of the peptide library (comprised of 175 unique phosphopeptides, 191 phosphorylation sites), where the vast majority were observed as 2+ ion species. We therefore interrogated a phosphopeptide-enriched cell lysate sample, showing that EThcD identified a substantially higher proportion of peptides with high confidence site localisation than HCD, irrespective of either the mass analyser used for MS2 or the search engine employed (Table 2).

**Table 2:** Proportion (percentage) of phosphosites localised with high confidence (<1% false localisation rate) with different fragmentation strategies (EThcD or HCD) using either high resolution (OT) or low resolution (IT) MS/MS data acquisition of a TiO_2_-enriched U2OS cell lysate. Data were interrogated using either Andromeda/PTM-score or MASCOT/ptmRS. Information was extracted from ref. 49

	Search algorithm	EThcD	HCD
Orbitrap (OT)	Andromeda/PTM-score	76	50
	MASCOT/*ptm*RS	86	76

Ion trap (IT)	Andromeda/PTM-score	76	37
	MASCOT/*ptm*RS	83	60

However, as observed with the peptide library, the slower EThcD duty cycle significantly compromised the total number of phosphopeptides identified (5733 *versus* 2413 for HCD and EThcD (Orbitrap MS2) respectively; equating to a reduction of ∼58%). Consequently, a higher number of phosphopeptides with high site localisation confidence (*ptm*RS score >99.0) were identified overall with HCD than EThcD (optimally using the orbitrap for MS2; 4337 *versus* 2078 respectively, an increase of 109%). These observations are somewhat more nuanced for ions of charge state ≥3+ where the MS2 acquisition strategy and search engine employed influence the choice of HCD or EThcD for optimal identification of confidently localised phosphorylation sites.

In an attempt to bypass the increased time requirements of dual fragmentation approaches, we explored a neutral loss-triggered regime that exploits phosphopeptide-derived loss of either 97.9763 Da and/or 79.9799 Da from the precursor ion in an initial HCD scan to trigger a second MS2 using EThcD.^49^ Theoretically, this approach benefits from the speed of HCD to define phosphopeptide precursors and minimises the effect of reduced scan rates on co-enriched unmodified peptides, while benefitting from the improved PTM-site localisation confidence observed with EThcD. However, upon implementation, we noticed a 12% reduction in the number of phosphopeptides identified using this neutral loss triggering approach, with only a small increase (3%) in the rate of correct PTM-site localisation *cf.* HCD alone (3873 *versus* 4337 phosphopeptides identified using HCD OT NL EThcD *versus* HCD respectively).^49^ Contrary to our expectations, we observed that ∼90% of confident phosphosite assignments were actually derived from the initial HCD scan rather than the secondary EThcD scan, concluding that our neutral-loss triggered EThcD method provided no additional benefit for this type of cell extract-derived phosphopeptide sample.^49^

Ultraviolet photodissociation (UVPD) is an alternative fragmentation method that has been successfully employed for characterising PTM-containing peptides, using high energy lasers at specific wavelengths to induce peptide fragmentation to generate all major types of product ions (a/x, b/y, c/z).^65^ The major advantage of UVPD is its highly optimisable parameters, with custom instrumentation being able to modulate wavelength, frequency, energy and irradiation times, as required for the sequence and charge state needs of the analytes.^65–70^ The Brodbelt and Heck groups have led developments in this area, demonstrating the benefits of phosphopeptide fragmentation with a (non-commercial) 193 nm UVPD laser. Their studies indicate higher phosphosite localisation confidence arising from the presence of ∼20–45% more phosphate-retaining product ions (*i.e.* no neutral loss) compared with HCD. However, as seen with EThcD, the increase in site-localisation confidence was offset by a lower phosphopeptide identification rate (<20%).^69,70^ Building on these investigations, we have investigated the effect of a commercial 213 nm UVPD set up (on the Fusion Lumos, Thermo Scientific) for fragmentation of a panel of phosphotyrosine (pTyr)-containing peptides. Our data broadly mirrored the phosphosite localisation confidence rates that were observed for the same analytes with HCD. However, we noted extremely poor fragmentation efficiency even with long irradiation times (∼100 ms); UVPD fragmentation at 213 nm was thus deemed impractical for complex samples.^71^ It also highlights the differences in spectral quality depending on laser set up.

The scientific interest in HTP phosphoproteomics studies has fuelled the development of multiple software packages for the interrogation of MS2 data that provide a measure of site localisation confidence, including *ptm*RS, AScore, PTM-score, LuciPhor and PTMprophet,^13,72–75^ each of which uses a different statistical strategy to infer confidence in site assignment. Although developers of these algorithms might suggest that true benchmarking is hard to perform ‘fairly’, our group and others have indeed attempted to do this from a user perspective.^49,72–74,76^ While there are caveats in terms of MS2 spectral resolution and fragmentation regime employed, in our hands *ptm*RS, in combination with the MASCOT search engine, overall provided the most confidently localised (and identified) phosphorylation sites when compared with AScore using either Andromeda or PEAKS as search engines.^49,72^ Interestingly, the more recently developed PTMprophet appears to outperform our optimal Mascot/*ptm*RS search strategy with respect to the number of confidently localised PSMs when using the same synthetic phosphopeptide library to our studies.^74^ A recent benchmarking study also encourages broad adoption of PTMprophet for phosphoproteomics data interrogation due to its ability to achieve higher sensitivity and quality.^77^ An interesting aspect of all these tools, and one which is consistently either not discussed or overlooked in almost all search algorithms, is the generation of chimeric spectra from isobaric phosphopeptide isoforms (where the same peptide sequence is phosphorylated on a different residue). Such peptides will likely co-elute and cannot be separated by *m*/*z*, resulting in co-isolation and generation of MS2 spectra that contain site determining ions from both species. This situation creates a paradoxical ‘coin toss’ scenario where evidence for confident localisation of both phosphosites within a single PSM results in statistical uncertainty. Consequently, site localisation algorithms typically report a 50% confidence for modification of each site, rather than reflecting the true 100% confidence that both sites are modified.

Incorporating ion mobility spectrometry (IMS) (drift tube IMS, FAIMS (high field asymmetric waveform IMS) or TIMS (trapped IMS)) in the LC-MS/MS phosphopeptide data acquisition pipeline has proven beneficial for the separation of such phosphopeptide isomers, improving their identification.^78–80^ Indeed, the recent study by Oliinyk and Meier using dia-PASEF demonstrated a substantive improvement in the identification of positional phosphopeptide isomers by TIMS, reportedly resolving 86% of the positional isomer pairs.^80^ Future improvement in IMS resolution, and routine incorporation of this conformation-based separation strategy into phosphoproteomics workflows could thus have a marked impact on the field.

## The challenges of non-canonical phosphorylation analysis

**Fig. 2:**
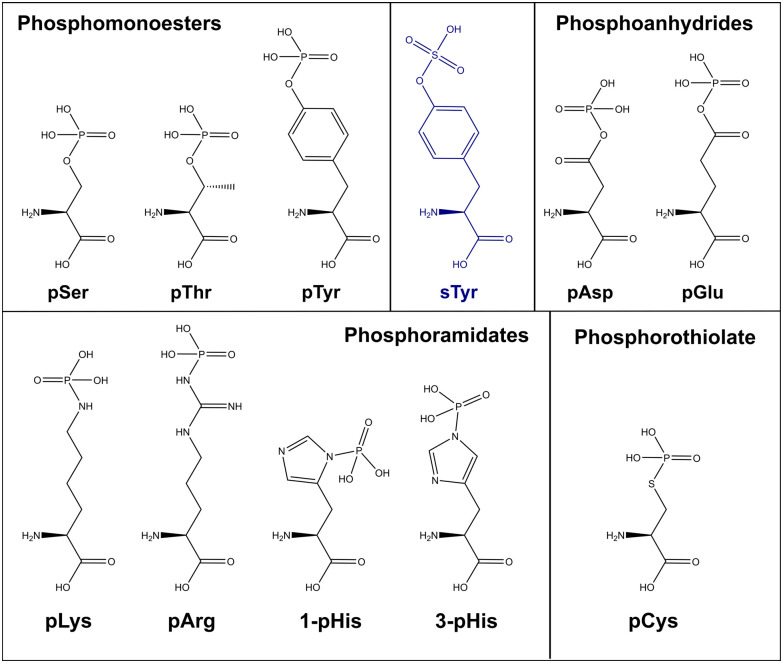
Chemical structures of biologically relevant phosphorylated amino acids, and sulfotyrosine (sTyr). The atomic structure of all canonical (phosphomonoesters – Ser, Thr, Tyr) and non-canonical (grouped by chemical property); phosphoanhydride (pAsp, pGlu), phosphoramidates (pLys, pArg, 1-pHis, 3-pHis) and phosphorothiolates (pCys) alongside sulfotyrosine (sTyr). All models retrieved from Chemdraw 20.0.

As discussed above, the field of phosphoproteomics has traditionally focused on the canonical phosphorylation of Ser, Thr and Tyr residues (referred to here as CPhos). However, another 6 amino acids can also be phosphorylated (referred to as non-canonical phosphorylation, NCPhos) (Fig. 2). These can be grouped into three classes based on their phosphate bonding chemistry: phosphorothiolates – Cys, conjugated through an S–P bond; phosphoanhydrides – Asp and Glu, conjugated though the hydroxyl (OH) of the side chain carboxyl group; phosphoramidates – His, Lys and Arg, conjugated through an N-P bond.^12,16–18^ Phosphohistidine (pHis) can also exist as two distinct phospho-isomers due to phosphorylation at either 1- or 3-position on the imidazole ring of the side chain (Fig. 2).^17^ Although phosphorylation of tryptophan is also chemically feasible, there is currently no evidence of this having been observed in biologically derived material (either as a free amino acid, or in a protein).^34^

**Fig. 3:**
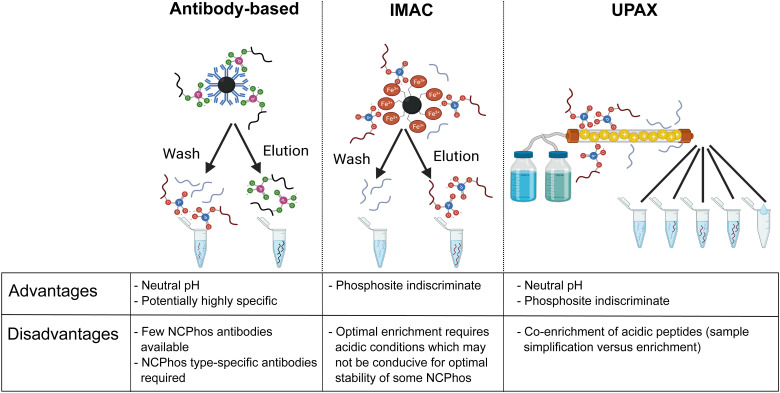
Strategies for the investigation of NCPhos. Table includes advantages and disadvantages of the three main enrichment strategies. Figure created in part with BioRender.com.

The biological relevance of NCPhos in prokaryotes is well documented, notably phosphorylation of His and Asp which play key roles in two component signalling (TCS) relays that mediate intracellular responses to environmental changes through receptor His kinases (extensively reviewed in ref. 81). Although NCPhos was first identified in mammalian cells over 60 years ago,^82^ there has been relatively little investigation of these PTMs, hence their involvement in regulating mammalian signalling remains somewhat controversial. One reason for the limited information on NCPhos, despite the number and scale of CPhos datasets available, is that they are not generally deemed to be of relevance and are therefore not considered during analysis. Phosphoramidates and phosphoanhydrides are also much less stable than CPhos (particularly under low pH or high temperature conditions), compromising enrichment strategies and sensitivity of analysis.^12,16–18^ Stability studies of pHis-containing peptides have shown that pH ≤ ∼2.5 results in extensive hydrolysis, with *t*_1/2_ at pH 1 (conditions as used for *e.g.*, TiO_2_ enrichment) being around 15 min.^12^ For perspective, phosphopeptide enrichment using standard protocols typically takes >1 h, hence compromising the use of such methods for investigation of NCPhos. Consequently, three broad approaches for enrichment of NCPhos (or pHis) have been developed (Fig. 3): antibody based, a modified IMAC approach and, our approach – UPAX (Unbiased Phosphopeptide enrichment using strong Anion eXchange).^12,83,84^

Antibody-based immunoprecipitation (IP) is often preferred as they can be (i) highly selective and (ii) used at physiological pH, minimising NCPhos hydrolysis. However, their application in HTP studies relies on the generation of an antibody that recognises the NCPhos residue in a sequence-independent manner, with each NCPhos also requiring its own antibody. The groups of Muir and Hunter have developed generic antibodies suitable for IP of pHis, both as general reagents and for isoform specific recognition; a site-specific antibody has also been generated for pHis18 within histone H4.^84–87^ Using their pHis isomer-specific antibodies, Fuhs *et al.* identified 786 pHis containing proteins from a human cell line extract using quantitative IP-based MS analysis. However, no specific pHis sites were defined.^84^ No other NCPhos antibodies are currently available, hampering the broad utility of antibody-based strategies for NCPhos discovery.

Large-scale pHis analysis has also been achieved using a rapid (14 min) Fe^3+^-IMAC-based enrichment strategy under relatively mild acidic conditions (pH = 2.3), demonstrating the feasibility of such approaches.^83,88,89^ This strategy successfully identified 276 sites of pHis on proteins from *E. coli*. However, application of this approach for the investigation of pHis sites from human cell extracts was met with relatively limited success: 83 acid-labile pHis sites were identified. Further filtering of the data based on the presence of a pHis immonium ion reduced this number to four.^89^ This relatively stringent filtering criterion may explain the disparity with the numbers of mammalian pHis-containing proteins as reported by Fuhs *et al.*, and observed in studies from our group. Although Leijten *et al.* report that 15–25% of the pHis peptides enriched from *E. coli* display an intense pHis immonium ion (being present in 13% of their synthetic pHis peptides), our evaluation, both of synthetic pHis peptides and pHis peptides from human cell extracts, suggests that the pHis immonium ion at *m*/*z* 190.04 is seldom observed with significant intensity.^12,83,88,89^ These differences could be explained by the fact that the relative abundance of any pHis immonium ion is likely dependent on the position of the pHis within the peptide sequence, as well as the normalised collision energy used for HCD.

Building on the application of this Fe^3+^-IMAC strategy for the enrichment of pHis-containing peptides, Lemeer and colleagues used this approach for large scale phosphoarginine (pArg) peptide enrichment from *Staphylococcus aureus*, permitting the identification of 470 ‘type I’ pArg sites (Andromeda localization probability >0.75), the largest cohort of pArg sites defined to date in any species.^90^ It is interesting that while this workflow could in theory be used for the interrogation of CPhos and NCPhos peptides (*i.e.*, considering all nine phosphorylated residues, assuming that acid labilities are similar), this is yet to be explored and it will be interesting to consider this approach in future.

Neutral pH IMAC-based systems, potentially capable of enriching all CPhos and NCPhos have also been reported. Whilst the utility of Phos-tag (an alkoxide-bridged dinuclear zinc(ii) complex)^91^ for NCPhos enrichment has not yet been investigated, SiO_2_@DpaZn has recently been shown to enrich phosphoramidate-containing peptides (pArg, pLys, pHis),^92^ albeit with co-enrichment of acidic peptides.

We have developed a strategy for the indiscriminate enrichment of NCPhos (and CPhos) along a similar vein, using (near) neutral pH to minimise phosphate hydrolysis. However, instead of using a bind/wash/elute bead-based approach, we developed a workflow exploiting strong anion exchange chromatography, termed UPAX.^12^ While we also observe binding of acidic peptides with UPAX, chromatographic fractionation following salt gradient elution simplifies the components of each fraction for analysis, compensating for the presence of these ‘unwanted’ peptides.

**Fig. 4:**
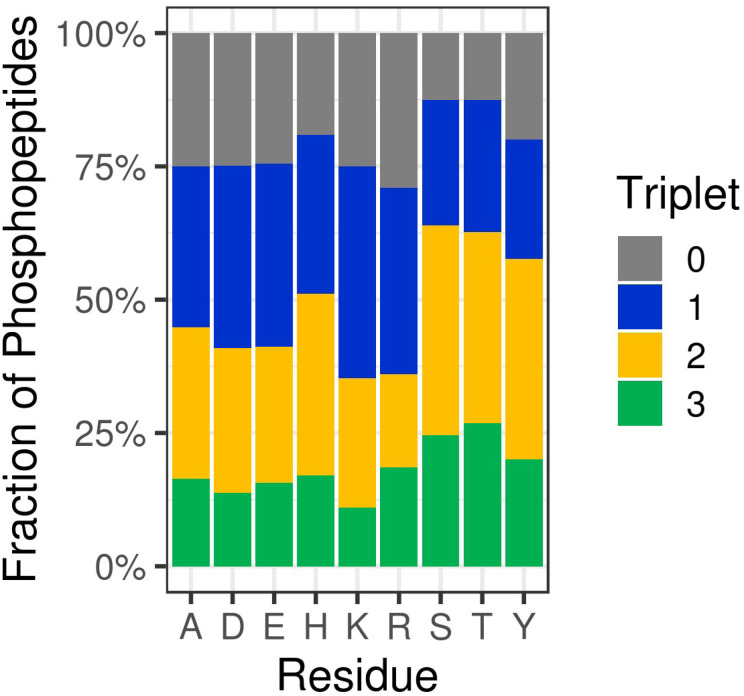
HCD-induced triplet neutral loss from different types of phosphopeptides. Percentage of phosphopeptides exhibiting no neutral loss (triplet = 0) or neutral loss of any combination of 80, 98 and/or 116 Da (Triplet = 1, 2 or 3) as a function of the defined site of phosphorylation following HCD (*ptm*RS >0.90). Ala (A) was used as a decoy for site localisation analysis. Figure adapted from ref. 12 with permission. Copyright © 2019 Hardman *et al.* Published by EMBO Press, under a CC-BY 4.0 license.

Somewhat unsurprisingly, we (and others) have shown that collision-induced fragmentation techniques (CID/HCD) induce extensive neutral loss from NCPhos-containing peptides, compromising the ability to generate site determining ions. To increase confidence in site localisation, or at least the type of amino acid to which the phosphate group is covalently bound, there have been a number of studies seeking to define residue-specific product ion features. Oslund *et al.* showed that pHis peptides subjected to resonant CID in a linear ion trap yielded predominant neutral loss of 98 amu from the precursor ion, and to a lesser extent, loss of either 80 amu or 116 amu (equating to loss of the phosphate group alone or with up to two water molecules), at the expense of backbone fragmentation.^93^ Whilst peptide identification thus required multistep activation (MSA), they were able to use this ‘triplet’ neutral loss feature (*Δ*80/98/116 amu) to discriminate pHis-containing peptides from those phosphorylated on Ser, Thr or Tyr. To avoid the need for MSA for identification and explore whether this triplet neutral loss pattern was observed for other NCPhos, we quantified HCD-induced precursor neutral loss across our UPAX-generated NCPhos dataset (*ptm*RS ≥ 0.90).^12^ Contrary to the strong triplet loss pattern observed with CID, we found that this neutral loss pattern was observed in less than 20% of pHis HCD spectra, with a similar proportion of spectra not showing any precursor ion neutral loss (Fig. 4). Of greater concern, spectra annotated as containing either CPhos or other NCPhos also yielded extensive triplet loss, indicating that for HCD, loss of 80, 98 and 116 amu from the precursor is not sufficient to define a phosphopeptide as being modified on His. As previously discussed, there is some disagreement in the field as to the prevalence of a pHis immonium ion that could be used for discrimination and positive identification of pHis. Although an in-depth analysis of immonium ion generation from NCPhos generally has yet to be performed, there are a small number of studies showing that immonium ions appear to be generated for pLys (at *m*/*z* 164.047 or *m*/*z* 181.074)^64,94^ and pArg (at *m*/*z* 237.0747 or *m*/*z* 209.0798), albeit with relatively low intensity and in an inconsistent manner.^90^ Hence, the use of immonium ions for CPhos or NCPhos discrimination currently remains controversial (possibly with the exception of pTyr^95^). EThcD thus remains the fragmentation method of choice for NCPhos identification, where a slight hit in sensitivity is compensated for by increased confidence in the nature (site and type) of the phosphopeptide identified.^12,64^

An important aspect when studying variable PTMs by MS-based proteomics and is particularly evident when considering nine different potential sites of phosphorylation, is the combinatorial expansion of search space as the number of PTMs under consideration is increased. This not only increases the required computing power but crucially, reduces the statistical power for peptide identification and PTM site localisation. To better understand the effects of increased search space for phosphosite localisation when considering CPhos and NCPhos, we developed a false localisation rate (FLR) estimation strategy, using a non-permissible phosphorylated amino acid (phosphoalanine, pAla) to define the rate of ‘random’ assignment of a phosphate group for each phosphorylated residue.^12,96^ This concept of using decoy amino acids to estimate site localisation confidence has since been expanded in a study that evaluated six different amino acids for their ability to act as suitable decoys for determining site localisation confidence. Using a variety of synthetic and complex biological sample-derived datasets, Ramsbottom *et al.* came to the conclusion that Ala (or potentially Leu) are the most appropriate decoys for reliable estimation of phosphosite FLR.^96^

Combining our UPAX technique for NCPhos (and CPhos) peptide enrichment from a U2OS osteosarcoma cell line extract with a neutral loss-triggered EThcD fragmentation strategy and our pAla decoy search approach, we were able to show that NCPhos is significantly more prevalent in mammalian cells than previously hypothesised, even when accounting for a relatively high (amino acid-dependent) false localisation rate.^12^ We identified approximately double the number of pAsp residues than seen for pTyr (the least frequent CPhos site) under the same conditions, with the frequency of pHis, pGlu, pLys and pAsp being marginally lower than pTyr. Far fewer sites of pCys were identified in this dataset. We have since validated one of the pCys sites identified in this HTP analysis, demonstrating that pCys412 on human PINK1 appears to be involved in regulating PINK1-mediated phosphorylation of ubiquitin.^97^ In a separate study, we have also shown that pCys90 on the transcription factor hypoxia-inducible factor (HIF)-1α is induced in hypoxia, hypothesising a role for this PTM in heterodimer formation.^27^ These data, combined with reports of a role for pCys as a Mg^2+^ sensor in PRL (phosphatases of regenerating liver) proteins^98^ speak to growing evidence of a role for pCys as a cell signalling regulatory element, beyond being ‘just’ a catalytic intermediate (as observed in *e.g.*, protein tyrosine phosphatase (PTP) 1B).^99^

A final point to note in considering confidence of identification and potential roles of CPhos and NCPhos is phosphate transfer; two component systems are known to operate through a phosphate relay mechanism from pHis to pAsp and there is evidence that such phosphate transfer can occur in the gas-phase during MS analysis. We have shown intermolecular phosphate transfer within a pHis-containing peptide dimer during resonant CID, generating a doubly phosphorylated product ion from a singly phosphorylated precursor.^100^ We believe that this is likely due to the nature of the system under investigation (high concentrations of a single peptide species) and the conditions used for fragmentation. Detailed investigation of a panel of synthetic pHis peptides subjected to HCD or EThcD failed to reveal any phosphate relocation.^12^ However, gas-phase re-arrangement during both CID and ETD has also been reported for pLys and pArg-containing peptides under certain conditions.^55,57,101,102^

Gas-phase rearrangement of peptide ions is not a new phenomenon; numerous groups have explored this during CID, both at the peptide level,^103–109^ and for CPhos.^54^ Although some studies have reported CID-mediated transfer of a phosphate group to an unmodified hydroxyl side chain in synthetic peptides,^54^ other studies on larger peptide cohorts have indicated that while this can be observed, the degree of phosphate transfer during CID is minimal and as such should not be of concern for HTP phosphoproteomics studies.^110^ Indeed, the phosphoproteomics community has certainly taken this to heart and the idea of gas-phase phosphate transfer is seldom mentioned. To our knowledge, almost all reports of gas-phase phosphate transfer are following resonant CID, rather than higher energy CID/HCD or EThcD as is more typically used today. Thus, while phosphate ‘jumping’ may be worthy of consideration in the future, we do not currently believe this to be a major source of misidentification in CPhos/NCPhos analysis. However, whether phosphate transfer contributes to NCPhos in mammalian systems *in vivo* remains to be explored.

## Sulfation, the forgotten +80 Da modification

An important yet largely neglected PTM is protein sulfation. Although first identified nearly 60 years ago,^111^ only ∼50 human proteins have been identified as ‘sulfated’ in UniProt, with the site of sulfation having been defined experimentally in approximately half of these. Current dogma suggests that this modification occurs primarily on Tyr residues as they transit through the Golgi and is thus found primarily on secreted proteins and those localised to the plasma membrane, regulating protein–protein interactions. The absence of known protein sulfatases suggests that Tyr sulfation is not reversible and its occurrence on residues that may be modified by numerous other types of PTMs (*e.g.* phosphorylation, nitration, adenylation)^112^ raises the possibility of site-specific PTM interplay, with sulfation preventing other regulatory modifications. Tyr sulfation is known to contribute to essential functions associated with host–pathogen interactions (*e.g.* HIV entry into cells), hormone/chemokine signalling, and pro-protein cleavage,^113–122^ somewhat explaining the extensive phenotypic abnormalities observed by knockdown/out of individual Tyrosyl-Protein-Sulfo-Transferase (TSPT) isoforms (infertility, hyperthyroidism, retinal function) and perinatal death following knockdown of both isoforms.^122^ However, experimental and computational studies indicate that up to 7% of Tyr residues in mammalian proteins may be sulfated, potentially making it the most abundant Tyr-based PTM. Assuming this is true, we are lacking a wealth of detailed information regarding ‘the where, the why and the what’ of this modification.^123,124^

From an analytical and phosphoproteomics perspective, sulfation is particularly important given the nominally isobaric mass difference of ∼9 ppm between sulfate and phosphate (Fig. 2). Consequently, it is possible to mis-identify sulfation as phosphorylation and *vice versa*, unless close attention is paid to the precursor *m*/*z* delta mass; it is also impossible to discriminate between these two PTMs with low resolution data.

**Fig. 5:**
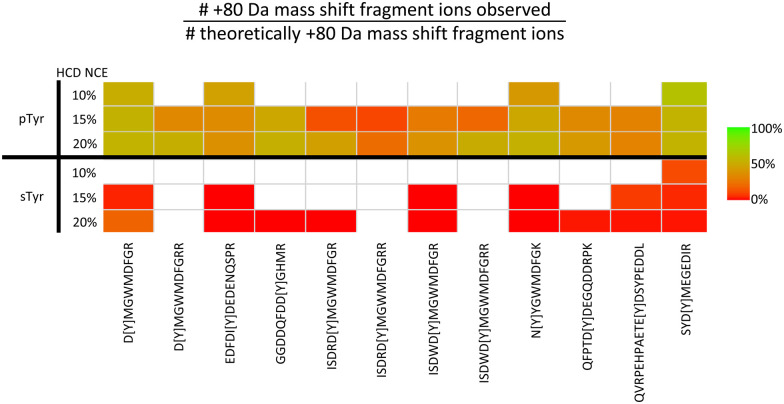
Challenges associated with localisation of sulfotyrosine (sTyr) *versus* phosphotyrosine (pTyr) on equivalent peptides following HCD. Identical sTyr- and pTyr-containing peptides were subjected to low NCE (10%, 15% or 20%) HCD and the degree of product ion neutral loss quantified using the equation. Green indicates all theoretical fragment ions contain the PTM induced +80 Da mass shift. Red indicates all theoretical fragment ions lack the +80 Da mass shift. White – no software based identification. Adapted from ref. 71 with permission. Copyright © 2023 Daly *et al.*, under a CC-BY 4.0 license.

Although sulfate and phosphate are both negatively charged, attempts to enrich sulfated peptides using well-established phosphopeptide enrichment strategies such as TiO_2_, anion exchange and IMAC (with Fe^2+^, Ga^3+^ and Ti^4+^ counterions) have so far not been very successful,^125,126^ hampering HTP sulfopeptide analysis. In recognition of this pressing need, we have recently developed a ‘sulfomics’ workflow. In-depth characterisation of 10 different IMAC counter-ions and TiO_2_ with a range of loading/wash solutions against a panel of sTyr (and identical pTyr) peptides allowed us to define conditions for preferential enrichment of sulfopeptides over phosphopeptides.^71^ In line with previous reports, we found that the recovery of sulfated peptides was minimal (<5%) with most of the IMAC counter ions investigated.^125,126^ However, using TiO_2_ or IMAC-Zr^4+^ in an acetic acid-based solution (importantly lacking glycolic acid, a common additive in phosphopeptide enrichment pipelines) we were able to demonstrate semi-preferential enrichment of sTyr peptides in a background of unmodified and phosphorylated peptides.^71^ As for CPhos and NCPhos-containing peptides, sulfated peptides exhibit extensive neutral loss, which is both more prevalent than seen for phosphopeptides, and occurs with both EThcD and HCD.^71,127,128^ Site localisation of sulfated peptides is therefore a major challenge. To understand the scale of this neutral loss and to define conditions optimal for the generation of site-determining product ions, we performed comprehensive fragmentation analysis of a panel of sTyr and pTyr peptides, using resonant CID, HCD, ETciD, EThcD and UVPD, at a range of different settings (a total of 43 conditions).^71^ These experiments revealed that any collisional dissociation resulted in near complete neutral loss of the sulfate moiety (80 amu) (Fig. 5), with a similar observation for UVPD. Although hybrid EThc/ciD approaches showed some potential for sTyr localisation, with up to ∼50% of product ions retaining the sulfate moiety, this was still much reduced compared with the equivalent phosphopeptide. Moreover, the reduction in the observed charge state of sulfopeptides *cf.* their phosphopeptide counterpart, meant that the efficiency of EThcD was peptide dependent.

Considering the CID/HCD-induced neutral loss propensity of sTyr, we evaluated the application of 10% NCE HCD for the discrimination of phospho- and sulfo-peptides. In contrast to the pTyr peptides where neutral loss (of either 80 or 98 Da) accounted for <1% of the total MS2 ion intensity, the equivalent sTyr peptides resulted in a median of 85% neutral loss (80 Da) with no evidence of neutral loss of 98 Da. Therefore, we postulated that using a neutral loss triggering strategy, where observation of −80 Da from the precursor at 10% HCD triggered a second MS/MS scan at 32% HCD, could enable ‘on-the-fly’ sulfopeptide identification, albeit in the absence of site localisation. In combination with our bespoke acetic acid-based enrichment protocol, and incorporating a phosphatase treatment step, we have now characterised 21 sulfated proteins (27 sites of sTyr) from a HEK293 cell secretome sample, a 70% increase in the number of identified sulfation sites. We think this HTP strategy shows great promise for future application across a range of biological systems and, while undoubtedly advancing the field, believe that there is still room for improvement to advance our understanding of sulfation as a PTM.

Given the significant increase in electronegativity of sY compared with pY (the primary p*K*_a_ of sY is −2.1 compared with 1.38 for pY) (as reported in the Human Metabolome database;^129^https://www.hmdb.ca), we suggest that the most promising avenue for confident localisation of sulfation sites in the future is likely to be negative ion mode MS. Such an approach has already been shown to increase ionisation efficiency and be less susceptible to neutral loss.^125,130,131^ However, HTP implementation would require development of suitable search algorithms for negative mode MS/MS data interrogation.

## Combinatorial PTM analysis by top-down proteomics

Unlike the peptide-based analyses detailed above, top-down proteomics (TDP) describes the characterisation of proteins as intact analytes.^132,133^ Rather than defining each modified peptide (or site) as an individual entity, losing understanding of the fine-tuning interplay of PTM connectivity at the protein level, TDP aims to define the complexity and heterogeneity of the various protein species that arise due to PTMs (covalent, chemical and cleavage), alternative splicing and single nucleotide polymorphisms (SNPs) (Fig. 1).^134^ Each of these myriad of protein forms, or proteoforms, likely has a unique biological function; their characterisation is thus essential to understand the true biological complexity of protein function and the role that SNPs and PTMs have on fine-tuning these processes.^2^

Unlike peptide-based analysis, interrogation of intact proteins in theory overcomes issues associated with missing peptides (compromising complete PTM mapping of proteins) and enables characterisation of PTM interplay on a single molecule, something which is not possible when considering modified peptides from a protein as separate entities.^135^ While bottom-up experiments benefit greatly from their ability to localise PTMs, crucial information on the overall proteoform population is lost. Given the prevalence of protein phosphorylation, its importance in biological regulation, and the priming function that specific phosphorylation events can play in mediating both other PTMs and biomolecular interactions, characterising the phosphorylation landscape (or indeed the PTM landscape) at the intact protein level is essential as we move to a genuine understanding of protein structure–function relationships. Hierarchical multi-site phosphorylation is an exquisite means of rapid and dynamic functional regulation which is not possible to define using peptide-based investigations.

Great strides have been made in TDP over the last quarter of a century. However, there remain substantial challenges, notably for accurate PTM site localisation on larger proteins (>∼30 kDa), and particularly when these modifications induce the same mass shift as is the case with multiple phosphorylation and/or sulfation events. High resolution mass spectrometers can be used to define the expressed gene product and identify the number and type of different PTMs based on MS1 spectra (mass determination) and a relatively limited number of MS2 product ions.^136,137^ Unfortunately, inefficient fragmentation and data analysis challenges, combined with the need to separate proteoforms with small mass differences (and even smaller *m*/*z* differences) means that we are still far from being able to achieve genuine proteoform characterisation for the vast majority of the proteome.

That being said, TDP studies are now capable of identifying tens of thousands of proteoforms from the thousands of proteins expressed in human cells.^3,137–139^ Yet these studies often fail to properly discriminate closely related proteoforms such as those that may differ by a single phosphate group, and are unable to differentiate those that are isobaric. Recent work in our group has focused on more targeted investigation of signalling proteins (>45 kDa) with the aim not only of defining the proteoform landscape (the number and type of PTMs), but critically, pinpointing the sites of modification, the aim being to define the hierarchical and combinatorial phosphorylation-mediated regulation of protein kinases needed to correlate proteoform with cellular function.^140^

**Fig. 6:**
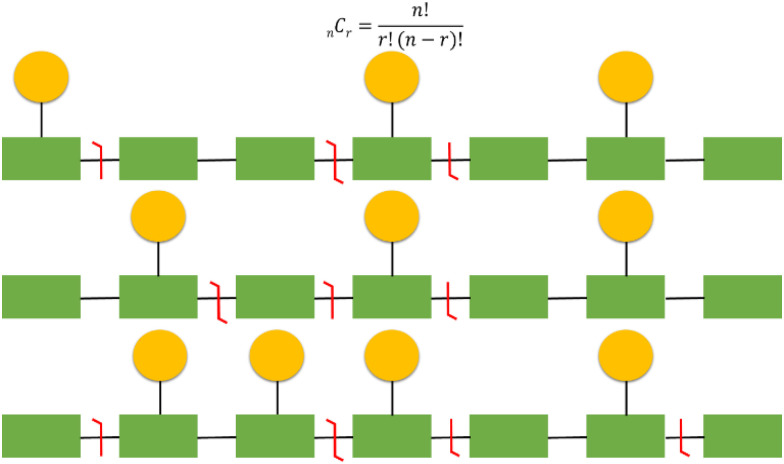
Challenges of proteoform identification and the need for site specific fragmentation to differentiate isobaric proteoforms. Depicted are examples of three different proteoforms (amino acids in green, phosphosites represented by yellow circles). Illustrating isobaric proteoforms (top 2) and proteoforms that differ by a single additional phosphate group (bottom 2). The equation can be used to calculate the number of potential proteoform combinations, where *n* is the number of potential phosphosites and *r* is the number of observed events.

It should be emphasised that localisation of covalent modifications and SNPs proves particularly challenging for larger analytes and those that are heavily modified due to an exponential increase in potential proteoform heterogeneity (Fig. 6). As a relatively simple but commonplace example, if a protein has 10 potential sites of phosphorylation and three phosphorylation or ‘action’ events, there are a theoretical 120 (_10_C_3_) combinations. Multiple versions of the triply phosphorylated protein may indeed exist within the cell at a given time, but it would be reasonable to assume that not all 120 combinations are biologically feasible or relevant. However, in a situation where multiple combinations potentially exist, comprehensive and confidently assigned fragmentation data is necessary for unambiguous proteoform identification. Hence, as protein size, and/or the number of action sites increases, this becomes even more challenging both from an analytical and a computational perspective. Moreover, the increased number of product ions generated as a function of protein size means that ion current is split, resulting in decreased signal to noise and compromising sensitivity.^141^

To compound these issues, protein fragmentation is generally not uniform; product ions preferentially arise from the protein termini, with fragmentation in the middle being more sporadic due to inherent retention of some higher order structure even under denaturing conditions – sometimes referred to as the ‘spaghetti’ model. PTMs are thus harder to localise if they lie within these central, less accessible regions.^142,143^ Current TDP search algorithms generally fail to highlight ambiguously localised PTMs, increasing the likelihood of misinterpretation in the absence of careful curation.

**Fig. 7:**
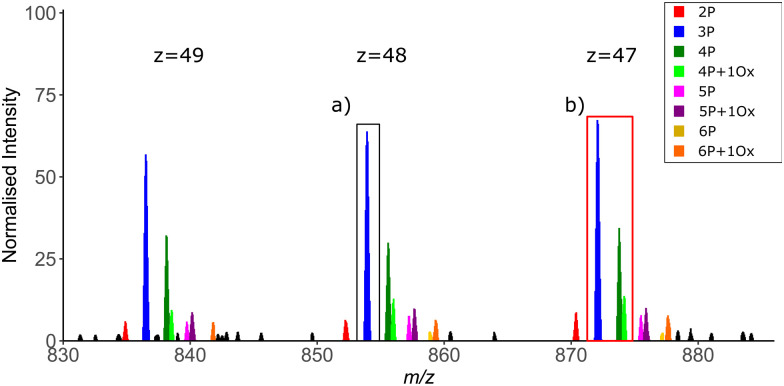
Intact mass spectrum showing proteoform complexity across three charge state envelopes of an exemplar ∼45 kDa protein. Depending on the occurrence of related proteoforms and the isolation width, it may not be possible to generate proteoform specific MS2 spectra, instead generating chimeric spectra from co-isolated species. Compare isolation of a single 3-phosphate containing ion ((a) black box) *versus* co-isolation of the multiple proteoforms including oxidised and non-oxidised variants of the 4 phosphate-containing species ((b) red box). *z* = charge state; P = phosphorylation; O = methionine oxidation.

As with any analyses, the manner in which the MS instrumentation is operated is fundamental to the quality of the data generated and thus the ease with which data can be interpreted. For TDP, scan resolution at both MS1 and MS2 levels is a critical feature. While isotopic resolution at MS1 level is preferred to allow charge state determination, the time taken to achieve this can be problematic on a separation timescale, *i.e* if MS measurement is coupled in-line with an LC system. Alternatively, charge deconvolution based on observation of different precursor ion charge states means that isotopic resolution is not essential to determine mass (Fig. 7). However, the greater the resolution at MS1, the easier it is to differentiate closely related proteoforms of the same species at high charge state, minimising precursor co-isolation and thus the generation of chimeric MS2 spectra. The width of the isolation window for ion selection for MS2 is also important in this context to minimise the number of potential conflicting site-determining ions within a given MS spectrum (Fig. 7).

In contrast, high resolution at the MS2 level (resolving power >50k) is necessary to allow deconvolution and thus assignment of the multiple overlapping product ions of different charge state.^144^ Averaging of multiple microscans for both MS1 and MS2 spectra can also improve the signal-to-noise ratio, albeit with a penalty on the overall duty time of the experiment.^141^

While advances in on-line proteoform separation strategies will undoubtedly improve site localisation capabilities in TDP (as proteoforms are introduced into the mass spectrometer at different times), the time taken for front end proteoform separation must be balanced against the requirement for extended duty cycles to maximise spectral quality.^145–147^

## Native IM-MS for the elucidation of PTM-induced conformational changes

While peptide and protein analysis under denaturing conditions can be used to elucidate information pertaining to the type and site of PTM (primary structure), it does not provide information on the effects of PTMs on higher order protein structure. X-ray crystallography and cryo-electron microscopy (cryo-EM) (and to a lesser extent NMR) remain the analytical methods of choice to study these effects given their ability for atomic level resolution. Although unable to yield such granular information, the relative ease of performing native ion mobility-MS (IM-MS) means that this technique has become an important component of the structural biologists’ toolbox.^148^ Numerous groups, including ours, have demonstrated the power of IM-MS to reveal changes in higher order structure (including PTM-mediated conformational dynamics, stability and oligomerisation state) and explore ligand-induced changes to structure (including *K*_D_ determination).^24,29,148–168^

**Fig. 8:**
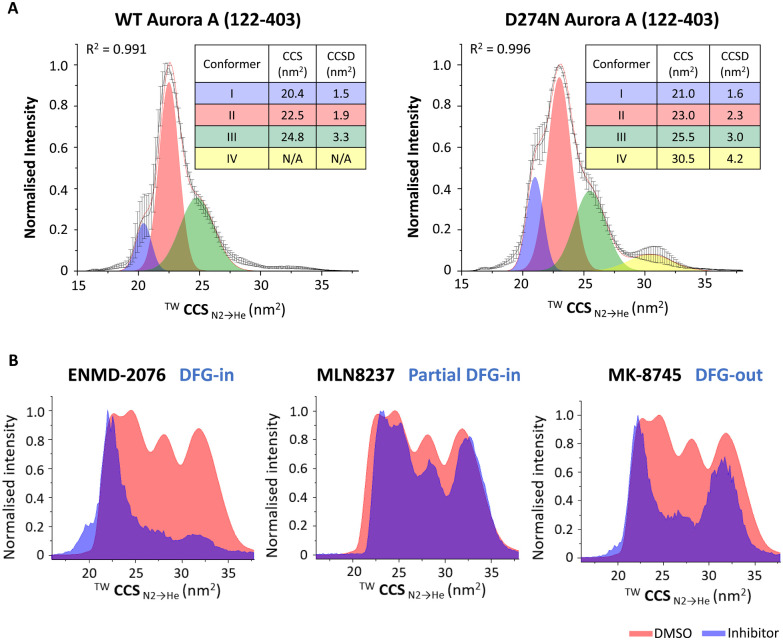
Exploring the effects of PTMs or ligand binding on the conformation of Aurora A (122–203). Native ion mobility mass spectra showing the ^TW^CCS_N_2_→He_ for the [M + 11H]^11+^ species of (A) (left) wild-type (WT) phosphorylated active Aurora A (122–403) and (right) non-phosphorylated inactive D274N Aurora A (122–403), detailing the rotationally averaged collision cross section (CCS) and CCS distribution (CCSD) indicative of conformer flexibility for each conformational state. (B) WT Aurora A in the absence (red, DMSO vehicle) and presence (blue) of inhibitors: ENMD-2076 (which favours DFG-in mode); MLN8237 (partial DFG-out); MK-8745 (DFG-out), following collision-induced unfolding with 26 V collision energy. Figure adapted from ref. 24 with permission. Copyright © 2022 Tomlinson *et al.* Published by American Chemical Society, under a CC-BY 4.0 license.

A number of studies have used IM-MS to investigate the effect of phosphorylation on structure, both at the peptide and the protein level.^29,79,150,169,170^ Some of our examples of the broad utility of native IM-MS include investigations into the protein kinases Aurora A, the catalytic subunit of protein kinase A (PKA_C_), and the NF-κB transcription factor p50.^29,150,171^ Building on our long-standing interest in the cell-cycle regulated protein kinase Aurora A, we used native IM-MS to define phosphorylation-induced protein conformational changes, determining that the weighted average collision cross section (CCS) for the phosphorylated, active version of this enzyme is larger than its non-phosphorylated, inactive (D274N) counterpart (CCS of 23.9 nm^2^ compared with 22.3 nm^2^ respectively). Furthermore, we demonstrated that the active (phosphorylated) protein existed as a smaller number of discrete conformers with smaller CCS distribution (CCSD), demonstrating that the phosphorylated active protein is more structurally constrained (less dynamic) (Fig. 8A).^171^ In comparable studies, we have shown that phosphorylation (or Ser to Asp phosphomimetic mutation) increases the CCS of both PKA_C_ and the NF-κB subunit p50 to a degree that exceeded the proportionally marginal increase in mass due to the PTM(s), while the effect on conformational flexibility (CCSD) appeared to be dependent on both the protein and the site of modification.^29,150^ Interestingly, our investigations of PKA_C_ showed conformational-dependent binding of PKI (the heat-stable inhibitor protein of PKA_C_), with preferential adduction to the more compact conformer and absence of binding to PKAc variants that lacked this specific conformer.^150^

Further Aurora A investigations also showed the utility of collision-induced unfolding IM-MS to distinguish binding modes of ATP-competitive inhibitors, revealing that different inhibitors ‘locked’ Aurora A in distinct partially unfolded inhibitor-bound states (conformers), correlating with previously reported classifications of Aurora A inhibitors as ‘DFG-in’, ‘DFG-out’ or ‘partial DFG-out’ (DFG-inter) (Fig. 8B).^171^

In a similar vein, a study by Nshanian *et al.* provided insight as to the mechanism of inhibition of the microtubule-stabilising tau protein by the ‘molecule tweezer’ compound, CLR01. In addition to identifying the CLR01 binding sites using top-down electron capture dissociation, they also used IM-MS to explore the role of phosphorylation on inhibitor binding, demonstrating marked compaction of the inhibitor bound 4R-tau isoform in a manner that was dependent on its phosphorylation state.^172^

While IM-MS investigations aimed at exploring the effects of PTMs and/or ligand binding on protein structure are still relatively limited, such studies highlight the benefits of this strategy to unravel the disparate effects of PTMs on protein configuration and their interplay with molecular or exogenous ligands.

## Final thoughts

The (apparently) never ending developments in the field of PTM analysis – separation strategies, technological solutions and computational algorithms, means that, as far as we have already come as a community in furthering our understanding of protein phosphorylation and other PTMs over the last century, there is plenty yet to explore. We are incredibly excited to see how continued analytical improvements advance our understanding of both phosphorylation and sulfation-driven biology. The scale of the challenge that we face means that as the community inevitably expands, and some analyses become more routine we must ensure that collectively we generate robust analytical data that is useful for advancing our knowledge on the effect of PTMs, alone or in combination.

## Author contributions

All authors contributed to writing – original draft preparation, review and editing of this manuscript. Claire E. Eyers: supervision, project administration; funding acquisition.

## Conflicts of interest

There are no conflicts to declare.

## Supplementary Material
